# The complete plastid genome of *Cotinus coggygria* and phylogenetic analysis of the Anacardiaceae

**DOI:** 10.1590/1678-4685-GMB-2021-0006

**Published:** 2021-08-02

**Authors:** Lingfeng Xu, Nong Zhou, Shunxin Zhao, Jingling Li, Xiaoying Pei, Jie Yu, Dongqin Guo

**Affiliations:** 1 Chongqing Three Gorges University, Chongqing, College of Biology and Food Engineering, Chongqing Engineering Laboratory of Green Planting and Deep Processing of Genuine Medicinal Materials in the Three Gorges Reservoir Region, China; 2 Southwest University, College of Horticulture and Landscape Architecture, Chongqing, China

**Keywords:** Plastid genome, genes, genetic resource, structure variation, phylogenetic analysis

## Abstract

*Cotinus coggygria* Scop. (Anacardiaceae) is an important ornamental tree with beautiful characteristics that is grown in China. In this study, the complete plastid genome of *C. coggygria* was sequenced and assembled. This genome was 158,843 bp in size and presented a typical tetrad structure, consisting of a large single-copy region (87,121 bp), a pair of inverted repeat regions (26,829 bp), and a small single-copy region (18,064 bp). A total of 134 genes were annotated, including 88 protein-coding genes, 38 tRNA genes, and 8 rRNA genes. We observed a deletion that caused the loss of the *rpl*32 gene, and a small expansion of IR regions resulted in the *trn*H gene accessing IR regions; two copies were obtained. Phylogenetic analysis showed that *C. coggygria* was most closely related to *Pistacia*, with 100% bootstrap support within Anacardiaceae. In this study, we report the plastid genome of *Cotinus* species for the first time, which provides insight into the evolution of the plastid genome in Anacardiaceae and promotes the understanding of *Cotinus* plants.

*Cotinus* is a small genus in Anacardiaceae and is mainly distributed in southern Europe, eastern Asia and temperate regions of North America (Matić *et al*., 2016). *Cotinus coggygria* Scop., commonly known as “smoke tree”, is usually considered either a large shrub or a small tree. This plant is cultivated in large urban parks, mountain scenic areas and gardens due to its brightly colored leaves and hardiness in barren soil (Miao *et al*., 2017). In late autumn, the leaves of *C. coggygria* are brightly colored and beautiful. In northern China, due to the cold climates, garden tree species are relatively monotonous and lack color. *C. coggygria* is the first choice for afforestation in northern landscapes or mountainous areas in China. The Fragrant Hills Park in Beijing has planted a large number of smoke trees, known as “fragrant hills red leaves”.

In this study, we sequenced and characterized the complete plastid genome of *Cotinus coggygria* and carried out a comparative study of Anacardiaceae plants. Our main analyses are as follows: 1) we sequenced and assembled the complete plastome sequences of *C. coggygria* for the first time; 2) the structural characteristics of *C. coggygria* plastomes were analyzed; 3) the boundaries of IR regions of plastomes were analyzed and described; and 4) the phylogenetic relationships of *C. coggygria* were inferred based on the complete plastome sequences. The results obtained here will provide a reference for the phylogenetic inference of *Cotinus* and studies on the evolution of plastomes in Anacardiaceae.

Fresh leaves of *C. coggygria* were collected from Mount Jinyun (geospatial coordinates: N29.842889, E106.394527), Chongqing, China. The samples were deposited in the Herbarium of Southwest University, Chongqing, with the accession number SWU-CQ2. Total genomic DNA was extracted using the CTAB method (Arseneau *et al*., 2017). The total DNA was ultrasonically fragmented. A DNA library with an insert size of 350 bp was constructed using the NEBNext® library building kit (Emerman *et al*., 2017) and was sequenced using the HiSeq Xten PE150 sequencing platform. Sequencing produced a total of 5.9 Gb raw data with 21,318,311 raw reads. Clean data were obtained using Trimmomatic (Bolger *et al*., 2014): we removed the low-quality sequences with more than 5% bases being “N”, and more than 50% of the total bases had a quality value of Q < 19. Ultimately, 21,200,559 clean reads were obtained after trimming.

*De novo* genome assembly from the clean data was accomplished utilizing NOVOPlasty v2.7.2 (Dierckxsens *et al*., 2017), with a k-mer length of 39 bp and a sequence fragment of the *rbc*L gene from maize as the seed sequence. A total of 286,982 reads were used in the final plastid assembly, and a circular genome was obtained. The average sequence coverage was 646. Bowtie2 (v2. 0.1) (Langmead *et al*., 2009) was used to ensure the correctness of the assembly by mapping all clean reads to the assembled genome sequence. The CPGAVAS2 (Shi *et al*., 2019) program was used to annotate the genome with the reference genome (*Pistacia chinensis*, GenBank: NC_046786.1). Apollo (Misra and Harris, 2006) was used to manually edit any annotations with problems. The genome maps were drawn in OGDRAW (Greiner *et al*., 2019). The genome sequence and raw sequencing data have been deposited in GenBank with the accession numbers MT876478 and SRR13076877.

The complete plastome sequence of *C. coggygria* is 158,843 bp in size and presents a typical tetrad structure, consisting of a large single-copy (LSC) region, a pair of inverted repeat (IR) regions, and a small single-copy (SSC) region, with lengths of 87,121 bp, 26,829 bp, and 18,064 bp, respectively. This structure is similar to those of other plants of Anacardiaceae. A total of 134 genes were annotated in the plastome of *C. coggygria* (Table S1, Figure 1), including 88 protein-coding genes, 38 tRNA genes, and 8 rRNA genes. We noted that the *rpl*32 gene was not annotated in this plastome. The *rpl*32 gene encodes a ribosomal protein and is the structural constituent of the 50S ribosome, which is considered to be involved in the biological process of translation (Weglöhner and Subramanian, 1993; Gaudet *et al.*, 2011). We retrieved the sequence of *rpl*32 from a closely related species (*Pistacia chinensis*) and performed a Bowtie 2 search on the complete raw data. Only 5 reads were partially mapped, and the sequence source of the unmapped part could not be identified after BLAST (Altschul *et al*., 1990) retrieval. We found no evidence to support transfer to the nucleus; therefore, we hypothesized that the deletion caused the species to completely lose the gene.

**Figure 1 -:**
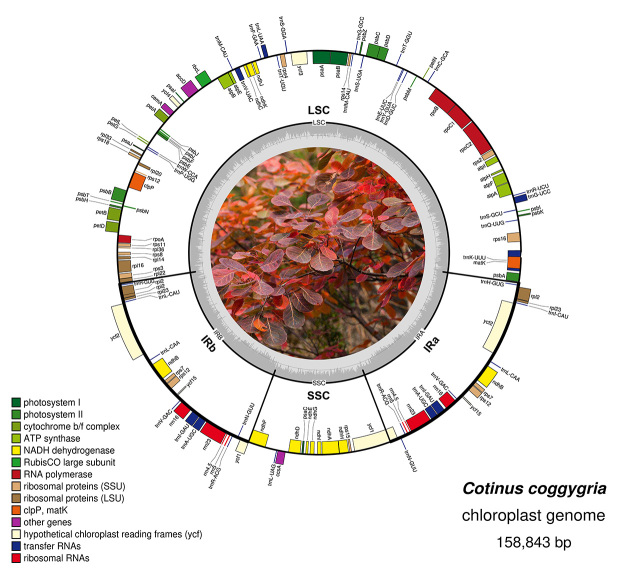
Graphic representation of features identified in the cp genomes of *C. coggygria* by using OGDRAW. The plastome has a conservative quartile structure composed of a LSC region, a SSC region and a pair of IR regions. The genes outside the circle are transcribed in counter clockwise direction, and the genes inside the circle are transcribed in clockwise direction. Different colors in genes represent different functions. The dark gray and light gray areas of the inner circle represent the GC content to AT content of the genome, respectively.

Simple sequence repeats (SSRs) were identified using the online website MISA (Beier *et al*., 2017), including mono-, di-, tri-, tetra-, penta-, and hexanucleotides with minimum numbers of 10, 5, 4, 3, 3, and 3, respectively. We detected 88 SSRs in the plastomes of *C. coggygria*. (Table S2). Most SSRs are mononucleotide homopolymers, particularly polyA (34) and polyT (35), which account for 88.75% of the total. In addition, there were 4 dinucleotides, 6 trinucleotides and 7 tetranucleotides.

The IRscope program (Amiryousefi *et al*., 2018) was used to visualize IR boundaries. The IR boundaries of *C. coggygria* and seven other taxa from Anacardiaceae were analyzed, and the results are shown in Figure 2. In the SSC/IRa boundary, the *ycf*1 gene spans this boundary and is mostly located in the SSC region, and it overlaps with IRa by 1097-1104 bp. *Spondias bahiensis* is an exception, and the overlap is 1401 bp. At the IRb/SSC boundary, *ycf*1 pseudogenes are produced by inverted repeats. Additionally, the *ndh*F gene crosses the IRb/SSC boundary (except for *Sclerocarya birrea*) and forms a partial overlapping region with the *ycf*1 pseudogenes. In general, there are no significant differences in SSC/IR boundaries.

**Figure 2 -:**
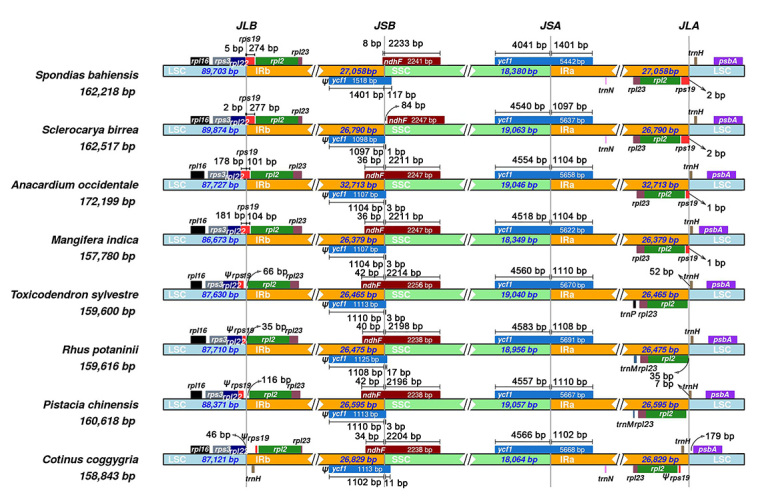
Comparison of the borders among LSC, SSC, and IR regions of eight analyzed species. The genes around the borders are shown above or below the mainline. The JLB, JSB, JSA, and JLA represent junction sites of LSC/IRb, IRb/SSC, SSC/IRa, and IRa/LSC, respectively.

At the LSC/IR boundary, we observed changes in the position of the *rps*19 gene in different taxa. We found that *rps*19 genes are completely located in the IR region in *Spondias* and *Sclerocarya* species; as a result, there are two complete copies in their genomes. In addition, the *rps*19 gene spans the LSC/IRb boundary and is partially duplicated in the IRa regions in *Anacardium* and *Mangifera* species. However, in *Toxicodendron*, *Rhus* and *Pistacia* species, we observed that the *rps*19 genes are located in the LSC region and are annotated as pseudogenes. Moreover, we observed a small expansion of IR regions in *C. coggygria* that resulted in the capture of the *trn*H and *rps*19 genes. The latter gene, *rps*19 in *C. coggygria,* is also a fragment and is annotated as a pseudogene. The rps19 gene encodes a ribosomal protein that is the structural constituent of the 40S ribosome, which is considered to be involved in the biological processes of translation and ribosomal small subunit assembly (Sánchez *et al*., 1996; Gaudet *et al*., 2011). Previously, *rps*19 was also observed to be a pseudogene in *Gentiana* species (Sun *et al*., 2018)*.*

To evaluate the divergence of plastomes among Anacardiaceae species, six plastomes (*C. coggygria*, *P. chinensis*, *R. potaninii*, *T. sylvestre*, *S. birrea* and *S. bahiensis*) were compared using shuffle-LAGAN mode in mVISTA (Brudno *et al*., 2003; Frazer *et al*., 2004) to identify interspecific variations. The reference annotation is *P. chinensis*. In addition, *M. indica* and *A. occidentale* were excluded from the analysis because it has been previously reported that their plastomes contain specific inversions in the LSC region or that there is a migration of DNA sequences from mitochondria in IR regions (Rabah *et al*., 2017). Therefore, they will not be discussed here. The IR regions are highly conserved compared to the SSC and LSC regions (Figure 3). Most of the regions with large divergence are observed in the intergenic regions, including *trn*H-*psb*A, *psb*M-*trn*D, *trn*T-*psb*D, *trn*T-*trn*L, *ycf*4-*cem*A, *psb*F-*pet*L and *ndh*F-*rpl*32-*trn*L. In particular, a deletion is present between the *ndh*F and *trn*L genes and results in the complete loss of the *rpl*32 gene in *C. coggygria*. Among the protein-coding genes, *rpo*C2, *rps*19 and *ycf*1 show high sequence differences.

**Figure 3 -:**
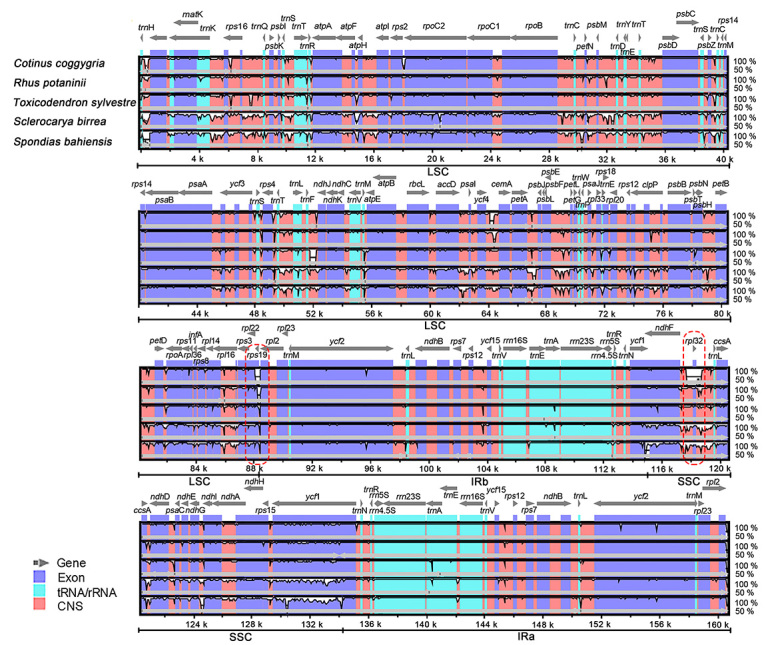
Comparison of the cp genomes in the six Anacardiaceae species by using mVISTA. The genes were represented as gray arrows on the top of the alignment. Different regions are labeled with different colors. The pink regions are "Conserved Non-Coding Sequences" (CNS), the dark blue regions are exons, and the light-blue regions are tRNA or rRNA. 50% and 100% refer to the similarity among sequences. Gray arrows above the aligned sequences represent genes and their orientation. In particular, two genes (*rps*19 and *rpl*32) are circled in red, as they are quite different in these taxa. The deletion of a large fragments in *C. coggygria* led to the loss of *rpl*32 gene.

To determine the phylogenetic position of *C. coggygria* in Anacardiaceae, we reconstructed maximum likelihood (ML) trees based on the plastome sequences. The plastid genome sequences of 16 species belonging to the family Anacardiaceae were downloaded from GenBank. Two Burseraceae species, *Boswellia sacra* and *Canarium album,* were used as outgroups. A detailed list of all plastid genomes analyzed in this paper is provided in Table S3. The complete plastid genome sequences were aligned using MAFFT online version 7.471 (Rozewicki *et al*., 2019). To avoid negative effects of sequence inversion and migration sequences from mitochondria on phylogenetic reconstruction, a total of 1,477 highly conservative blocks were identified using Gblock (Castresana, 2000; Talavera and Castresana, 2007) with the default setting. The Gblock alignment (138,527 bp) accounted for 71% of the original alignment (194,522 bp). These selected blocks were used to construct the phylogenetic trees separately using the maximum likelihood (ML) method implemented in RaxML (v8.2.4) (Stamatakis, 2014). The parameters were “raxmlHPC-PTHREADS-SSE3 -f a -N 1000 -m PROTGAMMALGX/GTRGAMMA -x 551314260 -p 551314260”. Bootstrap analysis was performed with 1,000 replicates. The results showed that all nodes had bootstrap support of 100%, indicating the reliability of phylogenetic recovery (Figure 4). In our phylogenetic trees, *Cotinus* and *Pistacia* were closely related. They belong to the tribe Rhoeae, together with *Rhus* and *Toxicodendron*. Unfortunately, few plastomes have been sequenced in Anacardiaceae, and we cannot describe the phylogenetic relationships of other Anacardiaceae plants in more detail based on plastome sequences. The application of NGS technology in Anacardiaceae plants is not sufficient; more plastome sequencing needs to be carried out in the future to improve the plastome resources of Anacardiaceae plants.

**Figure 4 -:**
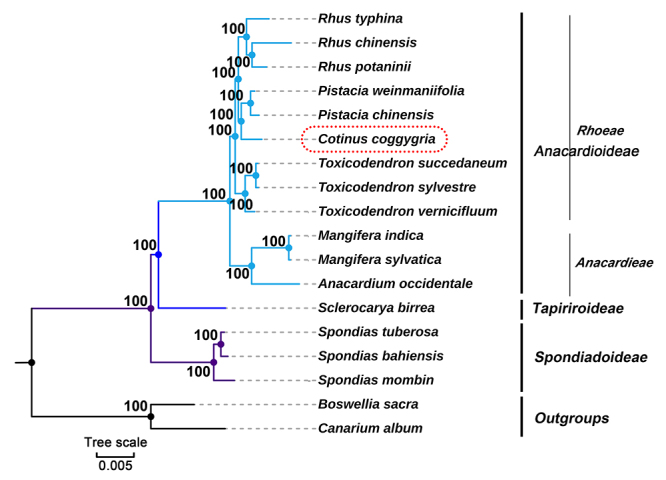
Phylogenetic relationships of species from Anacardiaceae family inferred using Maximum likelihood (ML) method. The phylogenetic tree was constructed by using the selected conserved blocks based on complete plastome sequences among the 18 plastomes. A total of 1,477 highly conservative blocks were identified by using Gblock with the default setting. Gblocks alignment (138,527 bp) accounts for 71% of the original alignment (194,522 bp). The number at the bottom of the scale, 0.005, means that the length of the branch represents the replacement frequency of bases at each site of the genome at 0.005. Bootstrap values were calculated from 1000 replicates. Two taxa from Burseraceae, namely, *B. sacra* and *C. album* were used as outgroups.

## Supplementary material

The following online material is available for this article:

Table S1 - Gene composition in the chloroplast genome of *C. coggygria*.

Table S2 - The Simple Sequence Repeats (SSRs) identified in the plastome of *C. coggygria*.

Table S3 - List of plastid genomes used for phylogenetic analysis.
