# Supplementary material for: The complete plastid genome of Cotinus coggygria and phylogenetic analysis of the Anacardiaceae
Source: Genet Mol Biol. 2021 Aug 2;44(3):e20210006. doi: 10.1590/1678-4685-GMB-2021-0006 (PMC8329748; doi:10.1590/1678-4685-GMB-2021-0006)
Supplement: Table S1 - [file 1415-4757-GMB-44-3-e20210006-s1.pdf]

Genetics and Molecular Biology
https://doi.org/10.1590/1678-4685-GMB-2021-0006

Supplementary Material to "The complete plastid genome of *Cotinus coggygria* and phylogenetic analysis of the Anacardiaceae"

Table S1 - Gene composition in the chloroplast genome of *C. coggygria*.

Category of Genes	Group of Genes	Name of Genes
Self-replication	Ribosomal RNA	*rrn*16S (x2), *rrn*23S (x2), *rrn*5S (x2), *rrn*4.5S (x2)
	Transfer RNA	*trn*A-UGC (x2)*, *trn*C-GCA, *trn*D-GUC, *trn*E-UUC (x3)*, *trn*F-GAA, *trn*G-GCC, *trn*H-GUG (x2), *trn*K-UUU*, *trn*L-CAA (x2), *trn*L-UAA*, *trn*L-UAG, *trn*M-CAU (x4), *trn*N-GUU (x2), *trn*P-UGG, *trn*Q-UUG, *trn*R-ACG (x2), *trn*R-UCU, *trn*S-GCU, *trn*S-GGA, *trn*S-UGA, *trn*T-CGU*, *trn*T-GGU, *trn*T-UGU, *trn*V-GAC (x2), *trn*V-UAC*, *trn*W-CCA, *trn*Y-GUA
	Large subunit of ribosome	*rpl*14, *rpl*16*, *rpl*2 (x2)*, *rpl*20, *rpl*22, *rpl*23 (x2), *rpl*33, *rpl*36
	DNA dependent RNA polymerase	*rpo*A, *rpo*B, *rpo*C1*, *rpo*C2
	Small subunit of ribosome	*rps*11, *rps*12 (x2)*, *rps*14, *rps*15, *rps*16*, *rps*18, *rps*2, *rps*3, *rps*4, *rps*7(x2), *rps*8
Photosynthesis	Subunits of ATP synthase	*atp*A, *atp*B, *atp*E, *atp*F*, *atp*H, *atp*I
	Subunits of photosystem II	*psb*A, *psb*B, *psb*C, *psb*D, *psb*E, *psb*F, *psb*H, *psb*I, *psb*J, *psb*K, *psb*L, *psb*M, *psb*N, *psb*T, *psb*Z, *ycf*3**
	Subunits of NADH-dehydrogenase	*ndh*A*, *ndh*B (x2)*, *ndh*C, *ndh*D, *ndh*E, *ndh*F, *ndh*G, *ndh*H, *ndh*I, *ndh*J, *ndh*K

	Subunits of cytochrome b/f complex	*pet*A, *pet*B*, *pet*D*, *pet*G, *pet*L, *pet*N
	Subunits of photosystem I	*psa*A, *psa*B, *psa*C, *psa*I, *psa*J
	Subunit of rubisco	*rbc*L
Other Genes	Subunit of Acetyl-CoA-carboxylase	*acc*D
	Translational initiation factor	*inf*A
	c-type cytochrom synthesis gene	*ccs*A
	Envelop membrane protein	*cem*A
	Protease	*clp*P**
	Maturase	*mat*K
Unknown	Conserves open reading frames	*ycf*1, *ycf*15 (x2), *ycf*2 (x2), *ycf*4
	Gene Fragments (pseudogene)	*ycf*1, *rps*19 (x2)

Note. (x) indicates that the gene has multiple copies, * and ** indicate that genes containing one/ two introns.
