# Supplementary material for: The complete plastid genome of Cotinus coggygria and phylogenetic analysis of the Anacardiaceae
Source: Genet Mol Biol. 2021 Aug 2;44(3):e20210006. doi: 10.1590/1678-4685-GMB-2021-0006 (PMC8329748; doi:10.1590/1678-4685-GMB-2021-0006)
Supplement: Table S2 - [file 1415-4757-GMB-44-3-e20210006-s2.pdf]

Genetics and Molecular Biology
https://doi.org/10.1590/1678-4685-GMB-2021-0006

Supplementary Material to "The complete plastid genome of *Cotinus coggygria* and phylogenetic analysis of the Anacardiaceae"

Table S2 – The Simple Sequence Repeats (SSRs) identified in the plastome of *C. coggygria*.

SSR nr.	SSR type	SSR	size	start	end	Location
1	p4	(TGAA)3	12	1425	1436	LSC
2	p1	(A)12	12	3460	3471	LSC
3	p1	(A)11	11	3627	3637	LSC
4	p1	(T)11	11	3835	3845	LSC
5	p1	(A)10	10	6631	6640	LSC
6	p1	(T)10	10	8726	8735	LSC
7	p1	(A)10	10	9145	9154	LSC
8	p1	(A)13	13	9553	9565	LSC
9	p1	(T)10	10	9908	9917	LSC
10	p1	(T)10	10	11193	11202	LSC
11	p1	(A)12	12	14202	14213	LSC
12	p1	(T)10	10	14225	14234	LSC
13	p1	(T)11	11	14306	14316	LSC
14	p1	(T)12	12	14318	14329	LSC
15	p1	(A)11	11	15708	15718	LSC
16	p1	(T)11	11	16604	16614	LSC
17	p4	(TTAT)3	12	16692	16703	LSC
18	p1	(T)11	11	19474	19484	LSC
19	p1	(T)11	11	19581	19591	LSC
20	p2	(AT)5	10	20954	20963	LSC
21	p1	(A)10	10	29102	29111	LSC
22	p1	(A)11	11	30221	30231	LSC
23	p3	(AAT)4	12	30351	30362	LSC
24	p1	(T)10	10	30862	30871	LSC

SSR nr.	SSR type	SSR	size	start	end	Location
25	p1	(A)10	10	31803	31812	LSC
26	p1	(A)11	11	31940	31950	LSC
27	p1	(T)12	12	33227	33238	LSC
28	p2	(AT)6	12	33555	33566	LSC
29	p1	(A)13	13	33775	33787	LSC
30	p4	(ATCT)3	12	38106	38117	LSC
31	p1	(A)10	10	38170	38179	LSC
32	p1	(T)11	11	38322	38332	LSC
33	p1	(A)10	10	38709	38718	LSC
34	p1	(A)10	10	47244	47253	LSC
35	p1	(A)10	10	47310	47319	LSC
36	p1	(A)10	10	47392	47401	LSC
37	p1	(T)11	11	49192	49201	LSC
38	p1	(T)10	10	49240	49249	LSC
39	p1	(A)13	13	49695	49707	LSC
40	p1	(T)11	11	51104	51114	LSC
41	p1	(T)11	11	51379	51389	LSC
42	p1	(T)10	10	51483	51493	LSC
43	p1	(T)10	10	56871	56880	LSC
44	p1	(A)19	19	57453	57471	LSC
45	p1	(T)10	10	61403	61412	LSC
46	p1	(A)10	10	62488	62497	LSC
47	p1	(T)11	11	63412	63422	LSC
48	p1	(T)10	10	66129	66138	LSC
49	p3	(TAA)4	12	67682	67693	LSC
50	p1	(T)12	12	67796	67807	LSC
51	p2	(TA)5	10	68013	68022	LSC
52	p1	(T)13	13	69385	69397	LSC
53	p1	(A)10	10	69790	69799	LSC
54	p1	(T)10	10	70147	70156	LSC

SSR nr.	SSR type	SSR	size	start	end	Location
55	p2	(AT)5	10	70790	70799	LSC
56	p1	(A)12	12	73629	73640	LSC
57	p1	(A)10	10	75304	75313	LSC
58	p1	(T)10	10	80922	80931	LSC
59	p1	(A)10	10	80949	80958	LSC
60	p1	(T)10	10	83140	83149	LSC
61	p1	(A)15	15	84208	84222	LSC
62	p1	(A)10	10	87145	87154	IRb
63	p1	(T)11	11	87516	87526	IRb
64	p3	(AAG)4	12	97069	97080	IRb
65	p1	(C)11	11	101431	101441	IRb
66	p1	(T)11	11	102505	102515	IRb
67	p1	(A)10	10	106331	106340	IRb
68	p1	(T)10	10	111965	111974	IRb
69	p1	(A)12	12	113807	113818	IRb
70	p3	(AAT)4	12	116158	116169	SSC
71	p4	(TTAA)3	12	116187	116198	SSC
72	p1	(T)12	12	116758	116769	SSC
73	p4	(TTGA)3	12	121279	121290	SSC
74	p4	(TTTC)3	12	123274	123285	SSC
75	p1	(A)11	11	128641	128651	SSC
76	p4	(CATT)3	12	129254	129265	SSC
77	p3	(TTA)4	12	129485	129496	SSC
78	p1	(A)11	11	129637	129647	SSC
79	p1	(T)10	10	130239	130248	SSC
80	p1	(A)10	10	131874	131883	SSC
81	p1	(T)12	12	132147	132158	IRa
82	p1	(A)10	10	133991	134000	IRa
83	p1	(T)10	10	139625	139634	IRa
84	p1	(A)11	11	143450	143460	IRa

SSR nr.	SSR type	SSR	size	start	end	Location
85	p1	(G)11	11	144524	144534	IRa
86	p3	(CTT)4	12	148885	148896	IRa
87	p1	(A)11	11	158439	158449	IRa
88	p1	(T)10	10	158811	158820	IRa
