# Supplementary material for: The complete plastid genome of Cotinus coggygria and phylogenetic analysis of the Anacardiaceae
Source: Genet Mol Biol. 2021 Aug 2;44(3):e20210006. doi: 10.1590/1678-4685-GMB-2021-0006 (PMC8329748; doi:10.1590/1678-4685-GMB-2021-0006)
Supplement: Table S3 - [file 1415-4757-GMB-44-3-e20210006-s3.pdf]

Genetics and Molecular Biology
https://doi.org/10.1590/1678-4685-GMB-2021-0006

Supplementary Material to "The complete plastid genome of *Cotinus coggygria* and phylogenetic analysis of the Anacardiaceae"

Table S3 - List of chloroplast genomes used for phylogenetic analysis.

Species	Family	Subfamily	Accession number
Anacardium occidentale	Anacardiaceae	Anacardioideae	NC_035235.1
Mangifera indica	Anacardiaceae	Anacardioideae	NC_035239.1
Mangifera sylvatica	Anacardiaceae	Anacardioideae	MN786795.1
Cotinus coggygria	Anacardiaceae	Anacardioideae	MT876478
Pistacia chinensis	Anacardiaceae	Anacardioideae	NC_046786.1
Pistacia weinmaniifolia	Anacardiaceae	Anacardioideae	NC_037471.1
Rhus chinensis	Anacardiaceae	Anacardioideae	MG267385.1
Rhus potaninii	Anacardiaceae	Anacardioideae	NC_049131.1
Rhus typhina	Anacardiaceae	Anacardioideae	NC_046837.1
Toxicodendron succedaneum	Anacardiaceae	Anacardioideae	MT211614.1
Toxicodendron sylvestre	Anacardiaceae	Anacardioideae	MT211615.1
Toxicodendron vernicifluum	Anacardiaceae	Anacardioideae	NC_046700.1
Spondias bahiensis	Anacardiaceae	Spondiadoideae	NC_030526.1
Spondias mombin	Anacardiaceae	Spondiadoideae	NC_035973.1
Spondias tuberosa	Anacardiaceae	Spondiadoideae	NC_030527.1
Sclerocarya birrea	Anacardiaceae	Tapiriroideae	NC_043919.1
Boswellia sacra	Burseraceae		NC_029420.1
Canarium album	Burseraceae		NC_048982.1
